# Biomimetic Polyurethane 3D Scaffolds Based on Polytetrahydrofuran Glycol and Polyethylene Glycol for Soft Tissue Engineering

**DOI:** 10.3390/polym12112631

**Published:** 2020-11-09

**Authors:** Kun Luo, Li Wang, Xiaohu Chen, Xiyang Zeng, Shiyi Zhou, Peicong Zhang, Junfeng Li

**Affiliations:** College of Materials, Chemistry & Chemical Engineering, Chengdu University of Technology, Chengdu 610059, Sichuan, China; luokun5373@163.com (K.L.); cxh15364965059@163.com (X.C.); zxy135475014426@163.com (X.Z.); zhangpeicong@cdut.cn (P.Z.)

**Keywords:** 3D scaffold, polyurethane, freeze drying, microstructure, soft tissue engineering

## Abstract

In this study, a novel polyurethane porous 3D scaffold based on polyethylene glycol (PEG) and polytetrahydrofuran glycol (PTMG) was developed by in situ polymerization and freeze drying. Aliphatic hexamethylene diisocyanate (HDI) as a nontoxic and safe agent was adopted to produce the rigid segment in polyurethane polymerization. The chemical structure, macrostructure, and morphology—as well as mechanical strength of the scaffolds—were characterized by Fourier transform infrared spectroscopy (FTIR), X-ray diffraction (XRD), scanning electron microscope (SEM), and tensile tests. The results show that the HDI can react mildly with hydroxyl (–OH) groups of PEG and PTMG, while gas foaming action caused by the release of CO_2_ occurred simultaneously in the reactive process, resulting in a uniform porous structure of PU scaffold. Moreover, the scaffolds were soaked in water and freeze dried to obtain higher porosity and more interconnective microstructures. The scaffolds have a porosity of over 70% and pore size from 100 to 800 μm. The mechanical properties increased with increasing PEG content, while the hydrophilicity increased as well. After immersion in simulated body fluid (SBF), the scaffolds presented a stable surface structure. The gas foaming/freezing drying process is an excellent method to prepare skin tissue engineering scaffold from PTMG/PEG materials with high porosity and good inter connectivity.

## 1. Introduction

In recent years, with the rapid development of medical technology, significant attention has been paid to the soft tissue engineering scaffold. Polyurethanes (PUs) have attracted much attention due to micro-phase structure composed of soft and hard segments, resulting in excellent mechanical properties and biological versatility [1,2,3,4,5]. Polyester is widely used in compositions of PU, however the materials with high strength and poor flexibility, which makes them not compatible for engineering soft and flexible tissue that can undergo dynamic loading, such as skin, tendons, ligaments, cartilage, blood vessels, and muscles [6]. To solve the above problems, researchers changed the composition and ratio of soft and hard segments to regulate the elasticity and biological function of PUs [7,8,9]. Although many related materials have been reported, the development of polyurethane elastomers with suitable mechanical and biological properties for soft engineering scaffold still has great demand.

Polytetrahydrofuran (PTMG) is a hydrophobic aliphatic saturated polyether elastomer and is used widely for the skin tissue engineering since the high flexibility and hydrolytic stability of molecular chains [10]. Obtained from tetrahydrofuran, which is a bioactive ether with high biocompatibility, PTMG is often used to synthesize block polymers [11]. Furthermore, the existence of PTMG can regulate the mechanical properties of the copolymer [12]. On the other hand, poly(ethylene glycol) (PEG) is widely researched and generally applied in soft engineering. PEG has excellent biocompatibility, non-toxic degradation, immunogenicity, and resistance to protein absorption [13,14]. PEG is an amphoteric polymer, can dissolve in many organic solvents, such as dichloromethane and tetrahydrofuran, as its ether can form hydrogen bonds with water [15]. Due to structure with two terminal hydroxyl end groups and the molecular chains having good ductility of PEG, it has strong water absorption and hydrodynamic radius. In the humoral environment, these properties provide an environment suitable for cellular infiltration and growth [16]. The hydrophilic character of PEG is a most important reason to apply it widely in the biomedical domain.

In addition to the influence of material composition for polyurethanes, synthetic preparation technology is also one of the important reasons. There are many methods for preparing polyurethane scaffolds, such as solvent casting/salt leaching, electrospinning, thermal phase separation, and gas foaming method. The pore size of the scaffold was controlled by controlling the size of the salt particles in solvent casting/salt leaching, but the connectivity of the material was reduced because of the uncontrollable removal of the salt particles in the later stage, which was not conducive to cell attachment and material transportation [17]. The electrospinning method can effectively control the fiber diameter and improve the mechanical properties of the material, but it is difficult to prepare the scaffold with suitable pore structure [18]. The thermal phase separation method can control the pore size by changing the preparation parameters but obtain higher porosity remains a challenge [19]. The porous scaffold with different pore structure and pore size can be obtained by controlling the rate and flow rate of gas escape for gas foaming method, which has good connectivity and simple preparation technology to avoid pollution [20,21]. With combination of these methods, better microstructure might be achieved.

In this work, porous and flexible polyurethane scaffolding for soft tissue engineering were developed, achieving good mineralization activity, surface wettability, and high elasticity. Thus, water soluble PEG and elastic PTMG were used as the soft segment, and HD was used as hard segment, while water was treated as a foaming agent. HDI was selected as the diisocyanate due to its aliphatic diisocyanate behavior and relatively non-toxic degradation by-product. The chemical composition, morphology, microstructure, mechanical properties, porosity, and wettability of PTMG/PEG porous scaffold materials were characterized and analyzed.

## 2. Experimental

### 2.1. Materials

Polytetrahydrofuran (PTMG, *M*_n_ = 2000 g/mol) and poly(ethylene glycol) (PEG, *M*_n_ = 2000 g/mol) was purchased from Jining Hua Kai Resin Co., Ltd, Jining, China. Hexamethylene diisocyanate (HDI, C_8_H_12_N_2_O_2_, >99.0%) and tin 2-ethylhexanoate (C_16_H_30_O_4_Sn, >95.0%) were obtained from Aladdin Chemical Reagent Factory, Shanghai, China.

### 2.2. Preparation of PTMG/PEG Porous Materials

**Figure 1:**
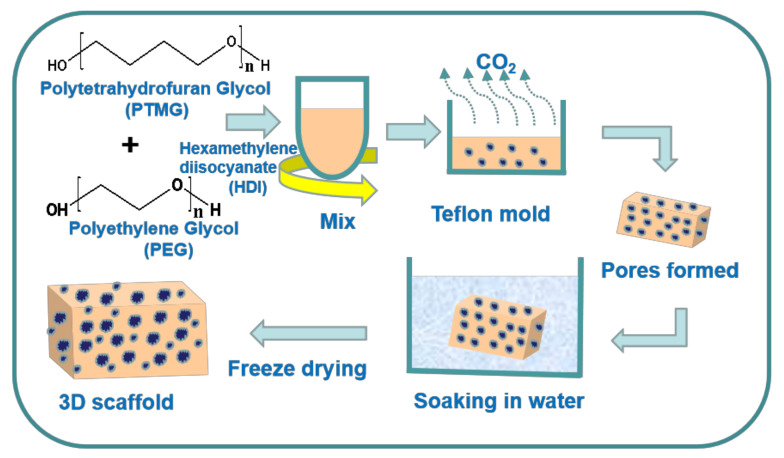
Preparation process of the polyurethane (PU) scaffolds.

PTMG and PEG were weighed and added into dichloromethane according to different PTMG/PEG ratios, respectively. The preparation process is showed as in Figure 1. The mixed solution was put in a 250 mL three-necked round bottom and synthesis was carried out under a dry nitrogen atmosphere while stirring at a temperature of 90 °C and a speed of 200–300 r/min. After stirring for 10 min, hexamethylene diisocyannte (according to the rate of hard and soft segment -OH:-NCO = 1:2) and tin 2-ethylhexanoate was added to mixed solution and reacted for 5 h. All processes were performed under a nitrogen atmosphere. Deionized water with 2% of the total system mass fraction and mixed solution were added into mold and cured over night at 120 °C in an oven accompanying with simultaneous foaming, thus rudiment of three-dimensional porous scaffold was obtained. For comparison, the same samples were prepared and freeze dried after being soaked in deionized water. The prepared porous scaffold materials were: PTMG/PEG 100/0; PTMG/PEG 75/25; PTMG/PEG 50/50; PTMG/PEG 25/75; PTMG/PEG 0/100.

### 2.3. Characterization

The crystalline structure analysis was carried out by conducted in the step size of 0.07° at a scanning speed of 0.3°/s with Cu-Ka radiation in the 2θ of 5–70° using an X-ray diffractometer (XRD) (DX-2700, China Fangyuan Instrument Co., Ltd., Wenzhou, China) operated at 40 kV and 25 mA. The microstructure morphology of the composite materials was analyzed by using a field emission scanning electron microscopy (FE-SEM, INSPECTF50, FEI, Eindhoven, Holland) at an accelerating voltage of 10kV. The samples were previously coated with gold in a sputtering device. Functional groups of PTMG/PEG materials were analyzed with a Fourier transform infrared spectrophotometer in a wave number range of 400–4000 cm^−1^ with 64 scans per spectrum at 4 cm^−1^ resolution. (FTIR, TENSOR-27, Bruker, Bremen, Germany). In addition, the thermal properties of PTMG/PEG porous materials were analyzed by differential scanning calorimetry (DSC) on a thermal analyzer (NETZSCH DSC-204F1, Selb, Germany) in a nitrogen atmosphere to measure the heat variation. First, samples were heated to 100 °C at a heating rate of 10 °C/min and hold at that temperature for 3 min to remove the thermal history, and then cooled to −50 °C, the second scan from −50 °C to 100 °C at 10 °C/min was operated.

### 2.4. Mechanical Properties, Porosity, Density, Water Absorption, and Contact Angle

The mechanical properties of porous composite materials were determined by an electronic universal testing machine (MIT-30KN, SFMIT apparatus Co., Ltd., Changzhou, China), including the compression properties and the Young’s modulus. Rectangular scaffold samples were made with measures of 10 × 10 × 10 mm and the loads were applied until the sample was compressed to approximately 60% of its original length. Following the guidelines of ASTM standard D695–96, five scaffold samples with the same composition were subjected to each test at room temperature, 65% RH and a crosshead speed of 1.0 mm·min^−1^ so as to gain an averaged value. The density, porosity, and water absorption ratio of porous materials were determined by a ceramic bulk density tester (DX-120C, Xiamen Qunlong Instrument Co., Ltd., Fujian, China) based on the Archimedes principle. Three parallel samples were tested simultaneously to calculate the average values. The results are calculated by the following formulas

Porosity (%) = (*M*_3_ − *M*_1_)/(*M*_3_ − *M*_2_) × 100% (1)

Water absorption ratio (%) = [(*M*_3_ − *M*_1_)/*M*_1_] × 100% (2)

Density (g/cm^3^) = (*M*_1_ × *D*_L_)/(*M*_3_ − *M*_2_) (3)

where *M*_1_ is the dry weight of the porous composite materials determined in air; *M*_2_ is the suspended weight of the anhydrous ethanol impregnated porous composite materials; *M*_3_ is the wet weight of the porous composite materials with saturated anhydrous ethanol state; *D*_L_ is the density of the saturated anhydrous ethanol.

The swelling behavior was evaluated the change of volume. As-prepared cubic samples with 10 × 10 × 10 mm (*V*_1_) to swell in deionized water, and then cured in a refrigerator with low-temperature, subsequently, the volume (*V*_2_) was measured after the sample was vacuum-freeze-dried. The results are calculated by the formula

Volume change ratio (%) = [(*V*_2_ − *V*_1_)/*V*_1_] × 100% (4)

In addition, PTMG/PEG porous scaffold materials were cut into thin slices were used to test the wettability and a minimum of three test pieces were used in each test. The water contact angle was measured by a contact angle meter (SDC 200, Yuding Precision Instrument Co., Ltd., Dongguan, China).

### 2.5. In Vitro Mineralization

Simulated body fluids (SBF, Na^+^ 142.0 mM, K^+^ 5.0 Mm, Mg^+^ 1.5 mM, Ca^2+^ 2.5 mM, Cl^−^ 103.0 mM, ${\mathrm{HCO}}_{-3}$ 4.2 mM, ${\mathrm{HPO}}_{4}^{2-}$ 1.0 mM, ${\mathrm{SO}}_{4}^{2-}$ 0.5 Mm, pH = 7.4) were used to assess the in vitro mineralization behavior of the porous composite materials. Each porous composite material was cut into small cubes (10 × 10 × 10 mm) and then were immersed in 25 mL of SBF and held in a water bath at 37 °C for 7 days. After 7 days of soaking, porous composite materials were taken out of the SBF and dried in a freeze-drier for subsequent observation by SEM.

## 3. Results and Discussion

### 3.1. Analysis of Fourier Transform Infrared Spectroscopy

**Figure 2:**
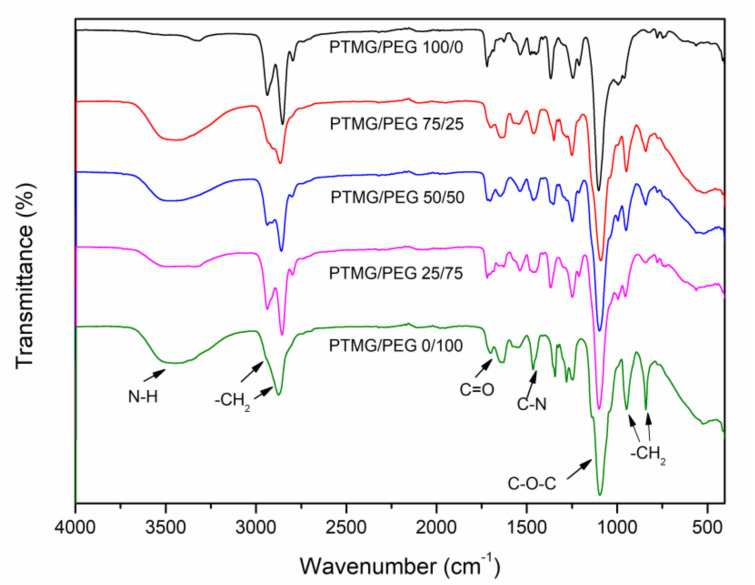
FTIR spectra of PTMG/PEG porous scaffold materials.

The FTIR spectra of PTMG/PEG porous scaffold materials with different ratios are shown in Figure 2. The broad absorption bonds from 3319 to 3530 cm^−1^ in the spectra belong to N–N stretching, while the peaks at 1637 and 1700 cm^−1^ are assigned to the stretching vibration C=O bond, and the C-N bond stretching was observed at 1465 cm^−1^, which indicates urethane combination bond formation in the polyurethanes [22]. The stretching vibration bond of NCO groups at 2250–2270 cm^−1^ are not observed, indicates that all HDI has reacted absolutely with PTMG and PEG [4]. The stretching vibration stand at 2862 cm^−1^ and 2934 cm^−1^ accorded with the asymmetric and symmetric stretching vibrations of the –CH_2_ groups, respectively. Furthermore, with increasing of PEG content in composites, the absorption peak area of –CH_2_ groups is increasing gradually at 840 cm^−1^ and 948 cm^−1^. These data indicate that the block copolymer PTMG/PEG porous scaffold materials were successfully developed.

### 3.2. Analysis of Crystalline Structure

**Figure 3:**
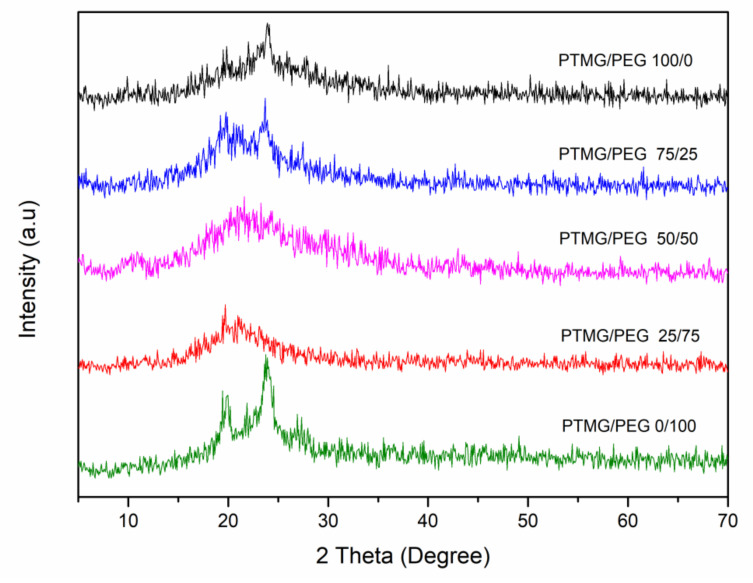
XRD of PTMG/PEG porous composites with different ratios.

The XRD patterns of PTMG/PEG porous scaffold materials with different ratios are shown in Figure 3. The diffractograms show that both PTMG/PEG 100/0 and PTMG/PEG 0/100 were semi-crystalline structure, with two low-intensity diffraction peaks at 20° and 23°. After copolymerization, PTMG and PEG became amorphous, with only a broad diffraction band nearby 21°. As both PEG and PTMG segments are with low molecular weight, which can reduce the crystallinity of the final product during crystallization [23]. Specifically, PTMG and PEG completely reacted with HDI formatting the cross-linked bonds in the soft hard segments, as a result, the original crystal structure of PTMG and PEG was destroyed, decreasing both the mobility of the segments and their crystallization capacity.

### 3.3. Thermal Properties

**Figure 4:**
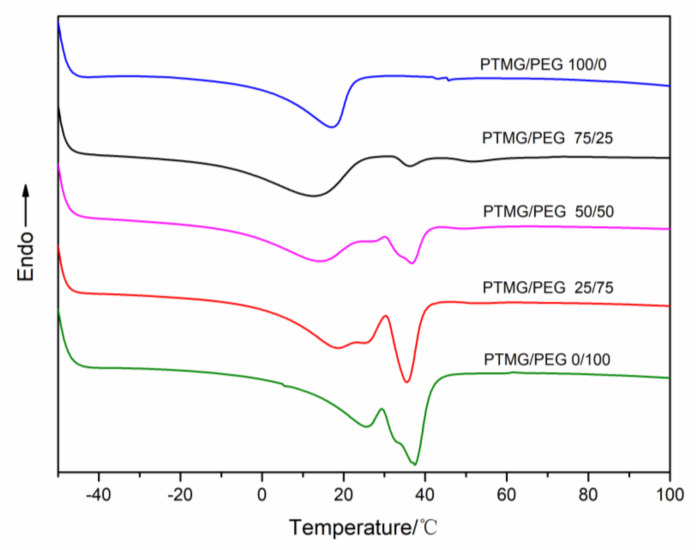
DSC of PTMG/PEG porous scaffold materials.

The thermal property of a polyurethane is affected by the raw materials, the proportions of the hard and soft segments, the density and type of crosslinking bonds, and the chain extender and synthesis method [24]. DSC was carried out to explore the thermal behaviors of PTMG/PEG porous materials (Figure 4). The crystallization temperature (*T*_cs_) and enthalpies (Δ*H*) and other thermal characteristic results are summarized in Table 1. The wider melting from −13 to 33 °C belongs to the PTMG segment, whereas melting peak at around 36 °C belongs to the PEG segment.

The crystallization temperature and Δ*H* of PEG portions increase with increasing the PEG content of the scaffolds, which could be interpreted by the existing PTMG segment in the copolymer that destructs the well-regulated arrangement of PEG chain segment. The influence becomes more obvious with further content of PTMG in the scaffolds. Accordingly, the melting peak area of PEG increases as the content of PEG in the scaffolds grows up. This is consistent with the XRD results. The amorphous porous scaffold material has a lower Δ*H* due to the irregular arrangement of molecular chains. Compared with the amorphous state of other ratios of porous scaffolds, the paracrystalline state of PTMG/PEG 0/100 is a primary reason for the higher crystallization temperature. 

### 3.4. Morphology and Microstructure

**Figure 5:**
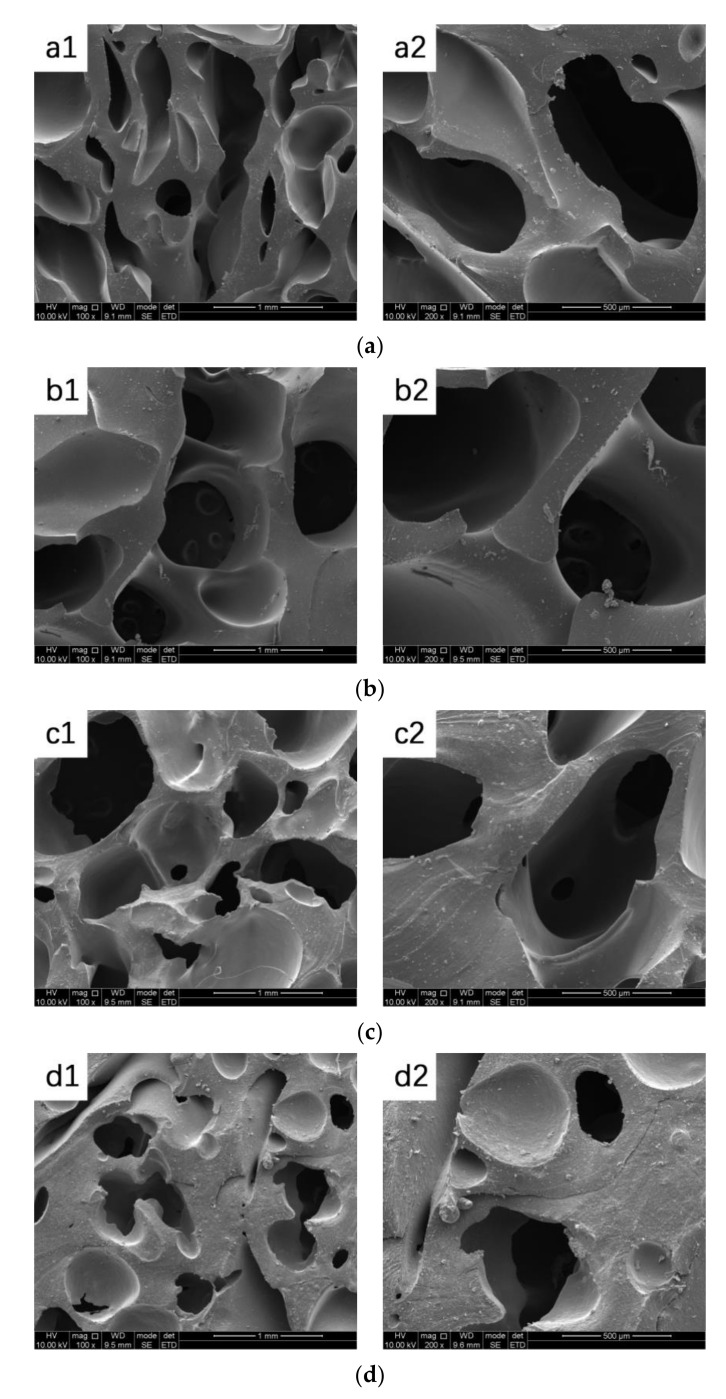

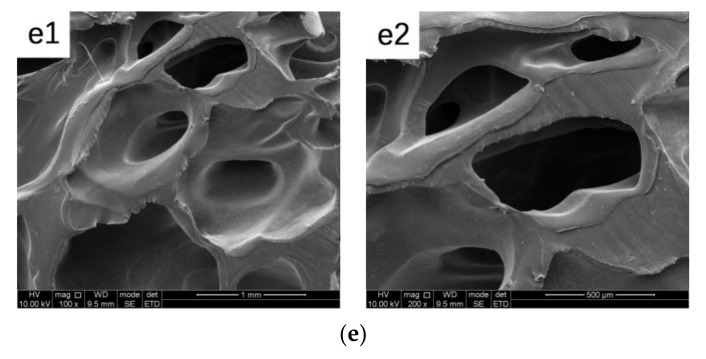
Scanning electron microscopy (SEM) images of (**a**) PTMG/PEG 100/0 (a1 100×, a2 200×), (**b**) PTMG/PEG 75/25 (b1 100×, b2 200×), (**c**) PTMG/PEG 50/50 ((c1) 100×, (c2) 200×), (**d**) PTMG/PEG 25/75 ((d1) 100×, (d2) 200×), (**e**) PTMG/PEG 0/100 ((e1) 100×, (e2) 200×).

The SEM morphology images of PTMG/PEG porous scaffolds was shown in Figure 5. All scaffold samples present high porosity and uniform pores. Smaller pore size of PTMG/PEG scaffolds range from 100 to 200 μm, while larger pore sizes between 400 and 700 μm are observed in all samples, showing a uniform pore distribution, and an excellent open-pore structure with predominant connectivity. The pore size of tissue engineering scaffold more than 100 μm is beneficial to transport nutrients and oxygen to increase cell viability [25]. The optimal pore size ranging from 200 to 600 μm promotes the infiltration and adhesion of cells and the formation of neovascularization [26].

The volume change before and after freeze-drying after soaking in water are shown in Table 2. Scaffold materials had a volume change ranging from 11.6% to 267.5%. The volume change of PTMG/PEG 100/0 was only 11.6%. As the PEG content in the scaffold increases, the volume change rate increases significantly, which could be interpreted by the molecular chain of the scaffold is stretched and expanded in saturated water, the original pore size increases, the three-dimensional pore structure expands, and the molecular chain is fixed in the extended state after low temperature treatment, and the change of pore structure can be observed by water evaporation of the scaffold after freeze-drying. Besides, the high-water absorption of PEG in scaffolds is one of the reasons for the large volume change. It was also worth noting that when the rate of PTMG/PEG scaffolds was 50/50, the volume change was 193.5%, which is due to the high porosity and flexibility of the scaffolds. The data of volume change are strongly conformed to the porosity and water absorption of scaffolds.

### 3.5. Mechanical Properties

**Figure 6:**
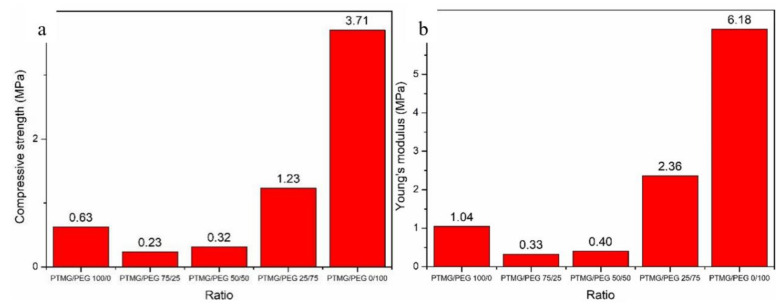
(**a**) Compressive stress (stain of 60%) and (**b**) Young’s modulus of PTMG/PEG porous scaffolds.

The mechanical properties of PTMG/PEG porous scaffolds were determined by compression tests. Figure 6 shows the compressive stress at 60% strain and Young’s modulus of PTMG/PEG scaffold materials. Mechanical properties are one of the most important factors for tissue engineering scaffolds, not only the growth of tissue and cell requires a certain strength, but also needs flexibility to adapt deformation [6]. According to the data, the compressive strength of PTMG/PEG 100/0 and PTMG/PEG 0/100 porous scaffold materials are 0.63 MPa and 3.71 MPa respectively. The compressive strength of the PTMG/PEG porous scaffold materials ranged from 0.23 to 1.23 MPa and the Young’s modulus was 0.33–2.36 MPa. It might be interpreted that the larger crystallinity of the PTMG/PEG 0/100 porous scaffold materials is good at mechanical properties, and the data of FTIR spectra is powerful evidence. With the content of PEG increasing, the compressive strength of scaffolds is strengthened, as well as Young’s modulus. The mechanical property of polyurethanes is concerned to several factors, such as crystallinity, the ratio of soft and hard segments, the porosity of scaffolds, etc. [4]. Then, the porosity of a porous structure is inversely proportional to its mechanical properties [27]. The results of mechanical properties conform to the porosity of scaffolds.

The data shows that PTMG/PEG 0/100 has higher compressive strength, but flexibility is also an important property for the organization. From the point of view of wettability, PTMG/PEG 50/50 has better water absorption and volume swelling rate, which shows that which is more suitable for the needs of tissue deformation due to its good flexibility in a body fluid environment. Besides, it can be seen from the micro morphology of the material that PTMG/PEG 0/100 has better pore structure and connectivity.

### 3.6. Water Absorption and Hydrophilicity

The porosity, water absorption and density of PTMG/PEG porous scaffold materials are shown in Table 3. The porosity of PTMG/PEG porous scaffolds was in the range from 74.9% to 89.5%. Porous scaffold materials had a water absorption ranging from 274.7% to 832.9%. The porosity of the material is affected by many factors, such as the content of foaming agent, the ratio of soft and hard segments and curing time. PEG has excellent water absorption, water can enter into the copolymer, so that the water can absolutely react with HDI to form a three-dimensional porous structure in the scaffold. PTMG/PEG scaffolds not only have suitable pore size and porosity to promote cell migration, tissue ingrowth, and nutrient transport but they also can retain the basic mechanical properties to support life activities. Furthermore, the content of PEG of scaffolds had a largely influence to water absorption. It was inferred that the existence of a large number of ether bonds in the PEG, and the oxygen atoms in the ether bonds form strong hydrogen bonds with the hydrogen atoms in the water, so it shows strong water absorption. It is noteworthy that the PTMG/PEG 50/50 had the maximum water absorption maybe since the high porosity can strengthen its water absorption.

**Figure 7:**
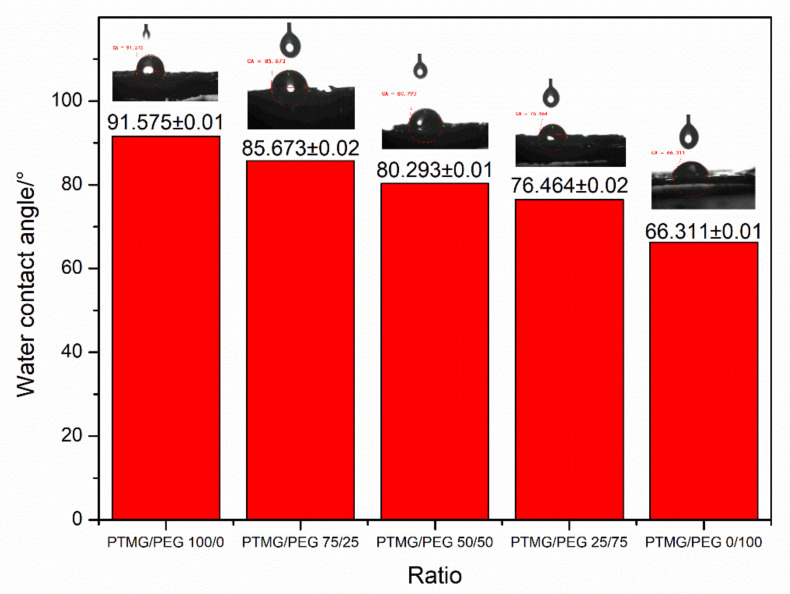
Water contact angle of PTMG/PEG porous scaffold materials with different ratios.

Figure 7 shows a histogram of average water contact angle and a photograph of the water contact angle of the scaffolds with different ratios. As to PEG has strong hydrophilicity, the water contact angles of the PTMG/PEG scaffold materials were decreased monotonically with the increasing contents of PEG within the range tested. All these indicated that the crosslinking network of PTMG and PEG can preferably improve the surface hydrophilicity and water absorption of scaffolds. Besides, the block polyurethane formed by the combination of PTMG and PEG through urethane bonds, while maintaining high hydrophilicity, also has good flexibility. In the swelling test of PTMG/PEG 50/50 porous scaffold material, it shows a volume change rate of up to 100%. It is beneficial that the porous scaffold material can better interact with soft tissues.

### 3.7. In Vitro Mineralization

**Figure 8:**
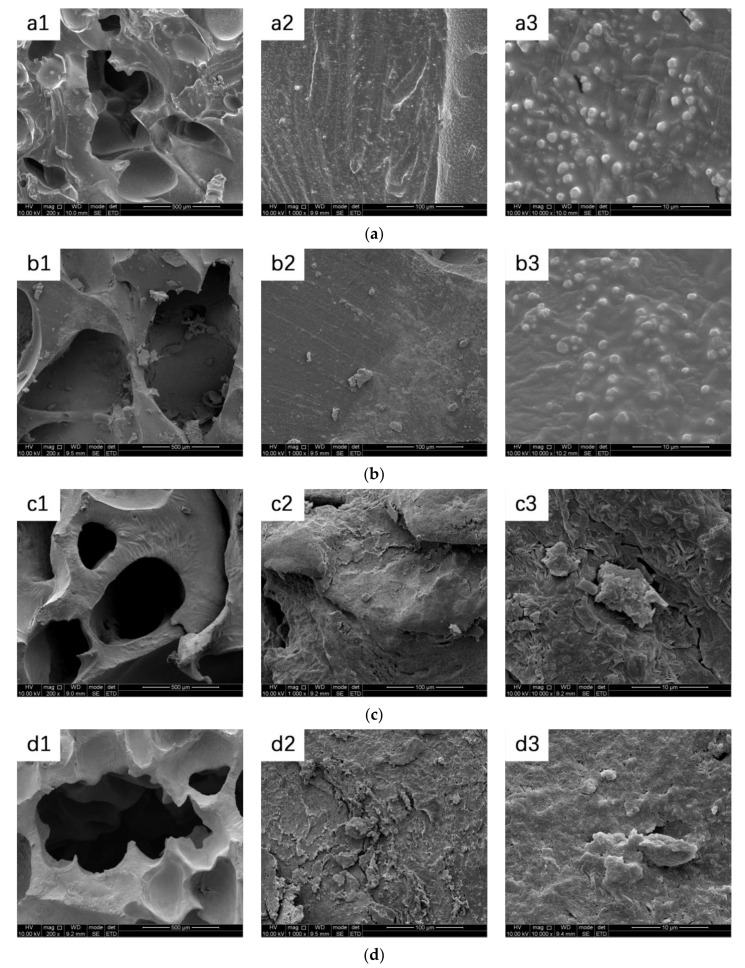

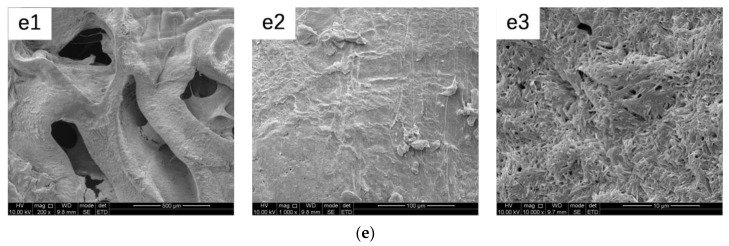
SEM images of (**a**) PTMG/PEG 100/0 (a1 200×, a2 1000×, a3 10000×); (**b**) PTMG/PEG 75/25 (b1 200×, b2 1000×, b3 10000×); (**c**) PTMG/PEG 50/50 ((c1) 200×, (c2) 1000×, (c3) 10000×); (**d**) PTMG/PEG 25/75 ((d1) 200×, (d2) 1000×, (d3) 10000×); (**e**) PTMG/PEG 0/100 ((e1) 200×, (e2) 1000×, (e3) 10000×) after 7 days of in vitro mineralization.

The ion concentrations of simulated body fluid (SBF) are nearly equal to those of human blood plasma [28]. The biologically active material can form apatite on its surface in the human blood environment and that this process can be reproduced in SBF. This means that the in vivo biological activity of a material can be predicted from the formation of apatite by mineralizing in SBF on its surface [29]. The surface of the porous scaffold materials showed visible changes after 7 days of SBF soaking (Figure 8). The mineralization of materials in SBF were observed, to evaluate the biological activity and bone inductivity of materials, and to significantly reduce the number and time of animal experiments [29]. New small pores appear on the scaffold surface. In addition, the surface of the PTMG/PEG 100/0 and PTMG/PEG 75/25 porous scaffold materials changed obviously after 7 days of immersion, and a large amount of deposition appeared on the surface of the material. It can be inferred that the scaffold induced apatite formation and deposited on the surface of the material during the immersion process. The other proportion of scaffolds did not observe particles in the surface obviously, and the surfaces were rougher with some small pores and cracks. It was interpreted that the curled molecular chain stretches after the material absorbs water and swells, and the pore structure of the material becomes larger after freeze-drying. Besides, the high wettability of porous materials lead to the sediments covering the surface of the materials rather than in the form of particles. Equations (5)–(7) stand for the reactions which may happen in the degradation process of polyurethane.

The ester groups were hydrolyzed into acids and alcohols

RCOOR′ + H_2_O→RCOOH + R′OH (5)

The carbamates were hydrolyzed into carbamic acids and alcohols

RNHCOOR′ + H_2_O→RNHCOOH + R′OH (6)

The urea groups were hydrolyzed into carbamic acids and amines

RNHCONHR′ + H_2_O→RNHCOOH + R′NH_2_ (7)

## 4. Conclusions

In this paper, a porous 3D copolymer scaffold from PEG and PTMG was developed by in situ polymerization and freeze dying, using HDI crosslinker. The results show that by combination of gas foaming and freeze drying, high porosity, and inter connective pores could be developed, resulting in a uniform porous structure of the PU scaffolds, with a porosity of more than 70% and pore size ranging from 100 to 800 μm. The mechanical properties increased with increasing of PEG, as well as the hydrophilicity. In conclusion, the gas foaming/freeze drying is a powerful method to develop PTMG/PEG scaffolds, which might be a potential option in soft tissue regenerative area.

## Figures and Tables

**Table 1:** Crystallization temperatures and enthalpies of PTMG/PEG scaffolds.

Sample	Onset Temperature (°C)		Offset Temperature (°C)		*T*_c_ (°C) ^a^		Δ*H* (J·g^−1^) ^b^	
	PTMG	PEG	PTMG	PEG	PTMG	PEG	PTMG	PEG
PTMG/PEG 100/0	−0.42	--	22.15	--	17.08	--	−32.9	--
PTMG/PEG 75/25	−12.57	32.82	24.24	40.7	14.42	36.09	−38.1	−1.7
PTMG/PEG 50/50	-8.8	28.99	27.14	40.31	14.05	36.86	−36.7	−9.81
PTMG/PEG 25/75	0.46	30.12	33.96	39.59	18.52	35.47	−42.8	−18.4
PTMG/PEG 0/100	7.16	27.07	33.98	41.62	25.53	37.48	−37.8	−31.3

^a^ Crystallization temperature from the 2nd heating run (*T*_c_); ^b^ enthalpy from the 2nd heating run (Δ*H*).

**Table 2:** Volume change before and after freeze-drying.

Sample	Dry Volume at Room Temperature (mm^3^)	Freeze Dried Volume with After Soaking in Water (mm^3^)	Rate of Volume Change (%)
PTMG/PEG 100/0	742.3	831.1	11.6%
PTMG/PEG 25/75	646.9	784.7	21.4%
PTMG/PEG 50/50	346.8	1021.6	193.5%
PTMG/PEG 75/25	230.3	557.6	144.1%
PTMG/PEG 0/100	433.6	1588.4	267.5%

**Table 3:** Porosity, water absorption, and density of PTMG/PEG porous scaffolds.

Samples	Porosity (%)	Water Absorption (%)	Density (g/cm^3^)
PTMG/PEG 100/0	76.1 ± 2.0	274.7 ± 24.3	0.3 ± 0.02
PTMG/PEG 75/25	74.9 ± 1.3	295.6 ± 20.4	0.2 ± 0.02
PTMG/PEG 50/50	89.5 ± 0.3	832.9 ± 32.1	0.1 ± 0.01
PTMG/PEG 25/75	83.8 ± 0.4	502.3 ± 10.2	0.2 ± 0.01
PTMG/PEG 0/100	85.1 ± 0.5	534.9 ± 23.1	0.2 ± 0.01
